# Colorimetric Ready-to-Use Microfluidic Chips for Rapid Antibiotic Susceptibility Testing of Methicillin-Resistant *Staphylococcus aureus* by a Smartphone-Based Analysis

**DOI:** 10.1021/acsomega.5c13266

**Published:** 2026-03-09

**Authors:** Cagla Celik Yoldas, Nilay Ildiz, Naim Yagiz Demir, Memed Duman, Erhan Yoldas, Sadik Kucukgunay, Kadir Erol, Ismail Ocsoy

**Affiliations:** † Department of Analytical Chemistry, Faculty of Pharmacy, 52966 Harran University, 63200 Sanliurfa, Turkey; ‡ Medical Imaging Department, Vocational School of Health Services, Bandirma Onyedi Eylul University, 10200 Balikesir, Turkey; § Department of Oceanography, Institute of Marine Sciences, Middle East Technical University, 33731 Mersin, Turkey; ∥ Nanotechnology and Nanomedicine Division, Institute of Science, Hacettepe University, 06100 Ankara, Turkey; ⊥ Department of Electrical and Electronics, Faculty of Engineering, Harran University, 63200 Sanliurfa, Turkey; # Department of Medical Pharmacology, Faculty of Medicine, 187470 Kirsehir Ahi Evran University, 40100 Kirsehir, Turkey; ¶ Department of Medical Services and Techniques, Vocational School of Health Services, 162313 Hitit University, 19030 Corum, Turkey; ∇ Department of Analytical Chemistry, Faculty of Pharmacy, Erciyes University, 38039 Kayseri, Turkey

## Abstract

Many phenotypic resistance tests have been developed for Methicillin-resistant *Staphylococcus aureus* (MRSA), but they still have disadvantages such as long incubation times, complicated procedure and a lack of portability. Herein, we designed ready-to-use microfluidic chips to use more advanced and new methods for analyzing antibiotic resistance specifically for the rapid detection of MRSA. Anthocyanin molecules, which change color depending on the pH value of the reaction medium in the microfluidic chip wells, are utilized as pH indicator in the test. The color change in the wells depends on the inhibition of antibiotic-resistant or susceptible bacteria at varying cefoxitin doses. The antibiotic resistance profiles of MRSA to multiple doses were revealed on a single chip. The obtained colorimetric responses were analyzed using a mobile application and color image processing techniques. The feasibility of the system was demonstrated using standard reference strains (*S. aureus* ATCC 43300 and ATCC 25923). Thus, this proof-of-concept study shows that antibiotic resistance detection can be performed in a few hours with minimal device requirements, warranting further validation with clinical isolates.

## Graphical abstract

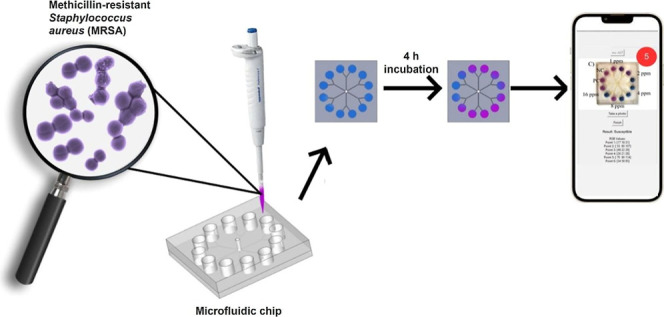

## 1 Introduction

Antimicrobial resistance (AMR) defined by the World Health Organization (WHO) is a serious problem that threatens the whole world.^1–3^ According to data from the US Centers for Disease Control and Prevention (CDC), there are more than 2.8 million infections associated with AMR and these infections induce more than 35.000 deaths in the USA every year.^2^ Methicillin-resistant *Staphylococcus aureus* (MRSA), classified as an antibiotic- resistant bacterium, is designated as a “High Priority” pathogen in the 2024 edition of the Bacterial Priority Pathogens List (BPPL) published by the WHO.^4^ MRSA infection causes various symptoms including endocarditis, local infection bacteremia and septic shock.^5,6^ Furthermore, it is estimated the patients infected with MRSA have a 64% higher mortality risk compared to individuals infected with methicillin-sensitive bacteria. The AMR can cause to prolonged hospitalization, increased healthcare costs, and life-threatening conditions.^7^

For these reasons, it is clinically important to treat and detect MRSA early to reduce its spread.^8–10^ For the diagnosis of MRSA in clinical laboratories, bacterial culture and liquid microdilution tests are performed to detect resistance profiles against methicillin/oxacillin antibiotics. However, these methods require time-consuming and complex procedures, and they need a lot of instruments and equipment.^11,12^ Because of these disadvantages, various tests have been developed to rapidly detect MRSA, such as enzyme-linked immunosorbent assay (ELISA) and polymerase chain reaction (PCR). These methods, which provide rapid results, are not widely used due to their high cost and time-consuming, complex sample preparation procedures.^13^ There is a still high demand to develop rapid, cost- effective, user-friendly point of care (POC) tests.

Microfluidic platforms have recently emerged as powerful alternatives to conventional phenotypic assays, transforming long time-consuming procedures into rapid diagnostic steps. These systems significantly reduce the workload, provide fast and accurate results, and require minimal sample volumes.^14,15^ In the specific context of antibiotic susceptibility testing (AST), various colorimetric microfluidic strategies have been reported to further simplify the readout. For instance, recent studies have utilized metabolic indicators such as resazurin within microwell arrays to monitor bacterial growth via smartphone-based color analysis.^16^ Similarly, comprehensive reviews have highlighted the integration of pH-responsive indicators and other optical sensors into microfluidic devices to accelerate detection times compared to conventional broth microdilution.^17^

Despite these advancements, many existing colorimetric platforms face limitations regarding quantitative standardization. They often rely on subjective visual inspection or complex, expensive optical readers (e.g., spectrophotometers) that negate the POC advantage. While color image processing using CIELab or Red Green Blue (RGB) algorithms^18^ offers a solution, issues related to lighting uniformity and device-dependent variability remain significant hurdles. Furthermore, recent smartphone-based approaches^19–21^ typically lack a fully integrated “closed-loop” system that combines standardized imaging hardware with a user-friendly decision algorithm for classification.

In this study, we address these gaps by introducing a novel, fully integrated microfluidic platform that combines the biocompatibility of anthocyanin-based sensing with a 3D-printed ratiometric smartphone interface. We developed a microfluidic chip-based methicillin resistance test that provides colorimetric results in 4 hours (h). The test solution contains medium, anthocyanin molecules and cefoxitin (cef). The test media loaded into the central input well provides the resistance profile against multiple doses of antibiotics after incubation. The test medium which was previously only available as a liquid,^22^ is now integrated into the microfluidic chip. This offers the advantage of fast results compared to multi steps processes. Bacteria that encounter antibiotics in the microfluidic chip continue their vital activities if they are resistant or are inhibited if they are susceptible. While the bacteria continue their vital activities, acidic organic volatile compounds (aOVCs) interact with the anthocyanin acted as a pH indicator in the test content and cause a color change. In the test we developed, this color change is analyzed semiquantitatively with a smartphone application. Unlike complex microfluidic systems that rely on external pumps and intricate valve networks, the proposed platform features a simplified, geometry-driven architecture. This deliberate design choice ensures pump-free operation and reduces fabrication costs, making it ideal for POC applications. Thus, a rapid, user-friendly, affordable and innovative test that contributes to the fight against AMR has been developed.

## 2 Experimental Section

### 2.1 Materials and Instruments

Tryptic soy agar (Merck, Germany), skimmed milk medium (Difco, USA), meat extract (Acumedia, UK), NaCl (Isolab, Türkiye), peptone (Mast Diagnostic, UK), cef (Merck, Germany) were all purchased from the companies indicated.

### 2.2 Microorganisms

MRSA standard strain 43300, Methicillin-sensitive *S. aureus* ATCC 25923 (MSSA) were obtained from Erciyes University, Faculty of Pharmacy, Pharmaceutical Microbiology research laboratory ATCC culture collection. All pathogens were stored in skim milk medium at −20 °C and regenerated prior to the experiments. The optical density was determined by spectrophotometer (Azure Ao, Azure Biosystems, Inc.).

### 2.3 Red Cabbage (*Brassica oleracea*) Extraction

Red cabbage (*B. oleracea* L., family Brassicaceae) is a source of anthocyanins that act as pH indicators in test media integrated into microfluidic chips. The red cabbage plant is rich in cyanidin-3-diglucoside-5-glucoside, an anthocyanin derivative, and is used as a pH indicator. For plant extraction, the purple-color leaves of the plant are separated, washed and dried. Then, 500 g of the leaves are cut into small pieces and boiled in 500 mL of distilled water for 30 min. The final purple-color extract is filtered with Whatmann No.1 filter paper to remove particles and impurities. It is stored in amber-color glass bottles. These are kept at 4 °C.^23^

### 2.4 Design of Microfluidic Chips

Two different microfluidic chips were designed in this study: 12-well with midchannel and 6- well with midchannel. The layered structures of the mid- channel chips (Figure 1A,D), from top to bottom, are as follows: 2 mm poly­(methyl methacrylate) (PMMA), 0.4 mm acetate sheet, 3 mm PMMA, and 1 mm PMMA. These layers were bonded using 0.13 mm thick double-sided tape (3M, High-Performance Acrylic Adhesive Transfer Tape 468MP). The wells and microfeatures in the PMMA and acetate sheet layers were fabricated using a CO_2_ laser machining system (AEON, NOVA 7). Each well has a diameter of 6.6 mm and an approximate height of 5.8 mm, including the double-sided tapes. The volume capacity of each well is approximately 200 μL.

### 2.5 Preparation of Colorimetric Ready-to-Use Microfluidic Chips

The liquid test medium integrated into the colorimetric microfluidic chips was prepared with minor modifications from reported studies.^22,24,25,35^ We briefly prepared a solution containing 10 g/L peptone, 1 g/L meat extract and 75 g/L salt to prepare the test content before loading it into the microfluidic chips.

The prepared solution was sterilized in an autoclave at 121 °C for 15 min. In the second step, the red cabbage extract was adjusted to pH 8.0 using a 1 M NaOH solution. Then, it was made sterile by passing it through a sterile filter. The autoclave-sterilized solution and sterile extract were finally mixed at a ratio of 1:4. Microfluidic chips are loaded with the antibiotics to prepare them for testing. For this, a cef solution prepared by serial dilution was added to the wells of the microfluidic chips as a positive and negative control and doses of 1, 2, 4, 8, and 16 μg/mL. The interpretation of resistance was anchored to the European Committee on Antimicrobial Susceptibility Testing (EUCAST) 2026 guidelines. Since the screening breakpoint for cef is defined as MIC > 4 μg/mL for *S. aureus*, survival and color change in microfluidic wells containing 4 μg/mL cefoxitin were considered indicative of MRSA. This design allows the assay to reliably distinguish between Susceptible (MSSA) and Resistant (MRSA) phenotypes, providing a binary qualitative classification suitable for rapid diagnostic decision-making rather than quantitative MIC estimation.^26^

Five μL of cef solution was added to each well and the chips were dried in an incubator at 37 °C. The test solution containing anthocyanin and the bacterial sample were mixed in a 1:1 ratio. We loaded the prepared solution through the central entrance hole of the incubator-dried chips. We added 2400 μL (for a 12-well chip) and 1200 μL (for a 6-well chip) of test solution at 200 μL per well. Following the inoculation, the microfluidic chips were incubated at 37 °C under stationary conditions (without shaking) for 4 h. To prevent the test solution from entering the channels or flowing back from the channels to the central inlet hole, to prevent evaporation and potential cross-contamination, the channels were filled with mineral oil and placed inside a humidified Petri dish. This setup ensured a stable reaction volume and prevented desiccation throughout the assay. Details regarding the storage conditions, long-term stability, and lot-to-lot reproducibility of the fabricated chips are provided in the Supporting Information (Exp. Section).

### 2.6 Digital Image Processing

Digital image processing was used to perform a semiquantitative analysis of the test results. At the end of the incubation period, the microfluidic chips were photographed using a white background. Color changes in the microfluidic wells were analyzed using ImageJ software (National Institutes of Health). ImageJ software was used to analysis the Red Green Blue values in the wells. With this data, the Red/Blue and Delta E values were calculated for the ratio of the blue-colored test to pink. Delta E, calculated according to the CIE 1976 Lab formula, shows the difference between the initial color and the end of incubation.^27,28^

1 ΔE=[(ΔL)2+(Δa)2+(Δb)2]12

In Formula [Disp-formula eq1], Δ*L*, Δ*a*, and Δ*b* are the key elements that define the dimensions of the CIE Lab color space. The difference between red and green is represented by Δ*a*, the difference between yellow and blue by Δ*b*, and the difference between black and white by Δ*L*. These values are low in similar images and high in different images.^29,30^ This analysis allows for more precise analysis of the color difference in the microfluidic chip compared to the human eye.

### 2.7 Statistical Analysis and Decision Rule

Semiquantitative analysis was performed by calculating the R/B intensity ratio and Δ*E* or Euclidean distance (ED) values for each microwell. To ensure data reliability and capture biological variability, the experimental design followed a rigorous replication structure. All experiments were performed as independent biological replicates on three different days (*n* = 3) using fresh bacterial cultures and separate microfluidic chips. Given the miniaturized format of the POC device, each antibiotic concentration was analyzed in a dedicated a single well on a 6-well microfluidic chip, and in two wells on a 12-well microfluidic chip. Consequently, the statistical evaluation focused on interchip reproducibility. Quantitative data are expressed as the Mean ± Standard Deviation (SD) of these independent replicates. The Relative Standard Deviation (RSD) was calculated to assess lot-to-lot consistency. To establish a standardized criterion for discriminating MRSA (high signal) from MSSA (low signal) in this proof-of-concept study, an analytical decision threshold (*T*) was defined based on the variation of the susceptible reference strain (MSSA ATCC 25923). Rather than performing a simple mean comparison which assesses population differences, we adopted a binary classification approach suitable for diagnostic assays. The cutoff value was calculated using the 3σ rule considering the principles of limit of detection (LOD) determination in analytical validation^31,32^

$$T=\mu_{susceptible}+{3\sigma }_{susceptible}$$

where μ_susceptible_ represents the mean R/B ratio and Δ*E* value of the susceptible strain replicates, and σ_susceptible_ denotes the SD. The decision was made using measurement results from wells containing 4 μg/mL and 8 μg/mL cef at the 4 h point. Statistical reporting focused on the analytical discrimination power. Samples yielding colorimetric signals exceeding this threshold (μ + 3σ) were considered analytically distinct from the susceptible baseline and classified as “Resistant”. To demonstrate method reproducibility and variability, semiquantitative data are presented as Mean ± Standard Deviation (SD), and error bars in all figures represent the SD of the replicates.

### 2.8 Development of a Smartphone Application

To minimize the need for a device in the color image processing step, we developed a smartphone application with image processing functionality in Python. The application can run on Android versions from 4 to 10 and does not require an Ethernet connection. The main menu consists of various interfaces for capturing, analyzing and evaluating the results of images. It calculates the RGB values of each well of the photographed microfluidic chips. Based on these values, the Euclidean distance (Formula [Disp-formula eq2]) is calculated. The result screen displays all RGB values and the Euclidean distance result, as well as indicating whether or not it is resistant.^33,34^

2 $${ED}^{2}={\left(R_{2}-R_{1}\right)}^{2}+{\left(G_{2}-G_{1}\right)}^{2}+{\left(B_{2}-B_{1}\right)}^{2}$$

The camera must be equidistant from the chips. It must not focus on other objects in the background. This will increase the accuracy of the test results. This is a significant challenge. Therefore, a smartphone platform was developed to standardize the distance between the camera and the microfluidic chips, provide balanced lighting and minimize environmental factors. Thanks to the platform, all tests are photographed and analyzed under the same conditions.

The mobile application employs a dual-parameter threshold algorithm to classify the antibiotic susceptibility status of the samples automatically. To minimize false positives and enhance diagnostic accuracy, the decision logic integrates both the ratiometric R/B intensity analysis and ED value. The classification thresholds were defined based on the statistical distribution of the susceptible MSSA strain replicates using the 3σ rule:

1. R/B Threshold (*T*_R/B_): μ_R/B_ + 3σ_R/B_
2. ED Threshold (*T*_ED_): μ_ED_ + 3σ_ED_

During the analysis, the app processes the captured image and calculates both metrics for each well. The sample is classified as “Resistant” (MRSA) if the signals exceed the respective thresholds; otherwise, it is reported as “Susceptible” (MSSA). This multimetric approach ensures that the diagnosis is validated by both the specific chromatic change (R/B) and the overall perceivable color change (ED).

### 2.9 Versatile 3D-Printed Smartphone Platform Design

We designed a smartphone platform to display color changes in microfluidic chips. Compared to our previous work, this platform has a more compact design.^35^ The platform is essentially made up of two main parts. The first is a stand for the smartphone, and the second is a drawer for the microfluidic chips. The drawer ensures the stability of the chips during photography and maintains a consistent distance between the camera and the chips for each analysis. The stand has an opening for photographing the chips in line with the phone camera. The stand contains a battery and a white LED light to support photography. The light is controlled by an on/off switch located outside the stand. The smartphone platform was fabricated using an Ultimaker S3 3D printer containing black, rigid poly­(lactic acid) (PLA) filament with a fill ratio of 20%. In this study, the design was changed to fit the Xiaomi Redmi 10S smartphone model. This was done by adjusting the microfluidic chip in the drawer and the phone camera aperture (Figure 6A).

## 3 Results and Discussion

The sensing mechanism of the microfluidic chips relies on the metabolic acidification of the culture medium by proliferating bacteria. During the rapid growth phase, bacteria metabolize carbohydrates with fermentation pathways, leading to the production of aOVCs. This accumulation of organic acids triggers a drop in the local pH of the wells. Consequently, the pH indicator (anthocyanin) undergoes a structural protonation, resulting in a visible color change from blue (basic) to pink (acidic). In the presence of effective antibiotics (cef), bacterial metabolism is inhibited, preventing acid production and maintaining the initial blue color.

The main components of the test are a naturally sourced anthocyanin used as a pH indicator and a culture medium. The anthocyanin molecules easily available to obtain, can be extracted directly from various plants especially from red cabbage (*B. oleracea*), is biocompatible, economical, and sensitive to pH changes.^36^ It has a wide color range in acidic, neutral, and alkaline environments. Unlike synthetic indicators, which appear in two different colors in acidic and basic environments, anthocyanin has a spectrum of five colors: pink, purple, blue, green, and yellow. The sensitive detection of aOVCs released by bacteria is possible owing to the wide color scale. Scheme 1 briefly illustrates the working mechanism of the microfluidic chip-based methicillin resistance test and its mobile application-based analysis.

**Scheme 1:**
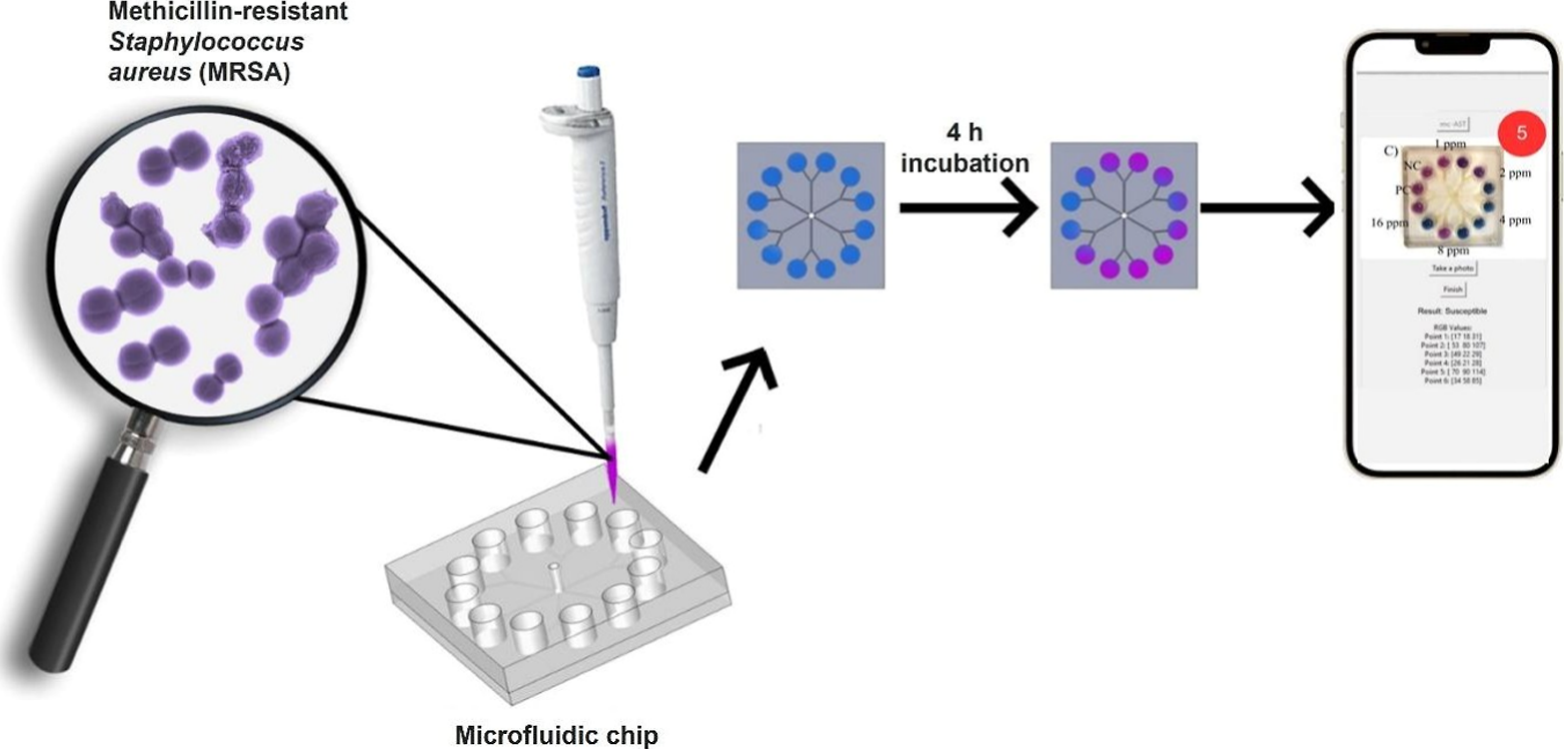
Schematic Illustration of MRSA by Digital Image Analysis

Previously, our team developed anthocyanin-based colorimetric tests to detect various bacteria including methicillin resistance ones.^22,24,25,35^ In current study, a methicillin resistance test was integrated into microfluidic chips, enabling the detection of multiple antibiotic doses in a single-step process. Figure 1 shows two different chip designs with 6 wells and 12 wells. Each well of the microfluidic chips contains a different dose of antibiotic. The bacterial sample sent from the central inlet of wells interacts with the antibiotics in the well. As a result of this interaction, susceptible bacteria to the relevant dose of antibiotic are inhibited. As a result of inhibition, the bacterium’s vital functions cease, and no aOVCs was released. If the bacteria are resistant to the antibiotic at the relevant dose, they continue its vital activities. Thus, they release aOVCs which make reaction environment acidic. The anthocyanin molecules are protonated due to pH changes, then initial test solution color, blue or purple, is turned to pink color As a result of these events the antibiotic dose dependent color change occurs simultaneously in each well. Two different microfluidic chips were designed to obtain the results mentioned above. Figure 1A,B show the layers and horizontal view of the 6-well microfluidic chip. Figure 1D,E show the layers and horizontal view of the 12-well microfluidic chip. Additionally, Figure 1C,F show the colorimetric responses obtained when the methicillin resistance test is integrated into both chips.

**Figure 1:**
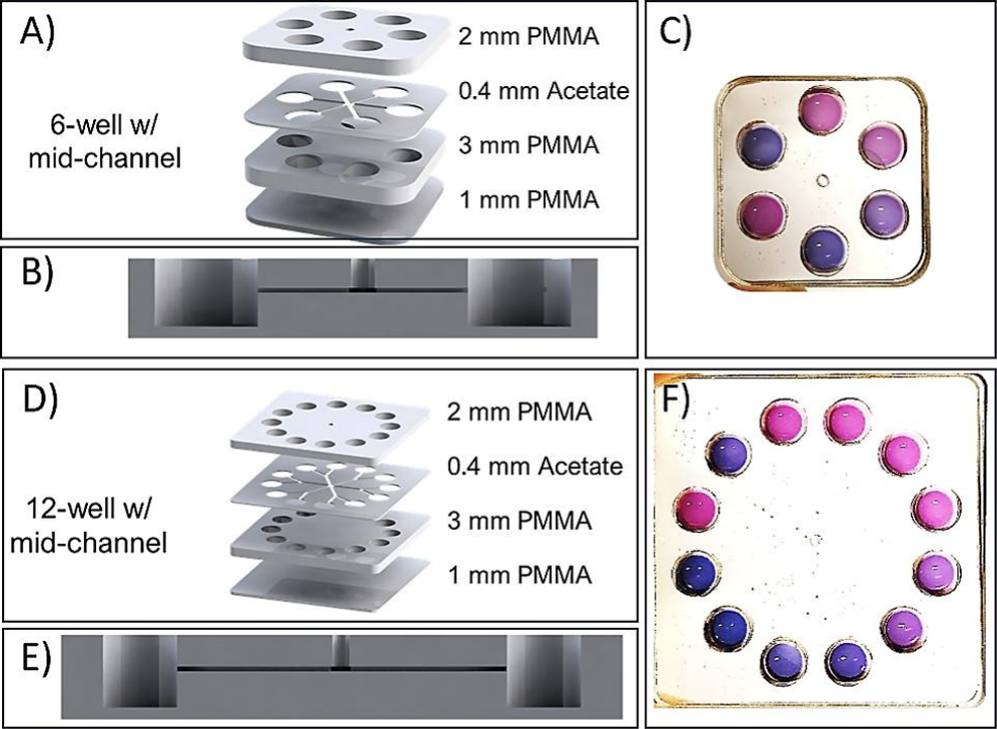
Graphical representations and images of the designed microfluidic chips. (A) Layers and (B) horizontal (cross-sectional) view of the 6-well microfluidic chip. (C) Colorimetric response obtained when the methicillin resistance test is integrated into the 6-well chip. (D) Layers and (E) horizontal (cross-sectional) view of the 12-well microfluidic chip. (F) Colorimetric response obtained when the methicillin resistance test is integrated into the 12-well chip.

In the first step, 5 μL of cef solutions at the doses indicated in the Figure (1–2–4–8 μg/mL) were added to each well of the 6-well microfluidic chips and dried. In Figure 2, 600 μL of anthocyanin test solution (pH 8, 1:4 anthocyanin ratio) was mixed with 600 μL of 3 McFarland bacterial solution, and a total of 1200 μL of anthocyanin test solution was loaded into the center inlet of each well. The negative control contained 32 μg/mL cef, and the positive control contained 0 μg/mL cef. MRSA was tested in Figure 2A, and MSSA was tested in Figure 2B. In Figure 2A, all wells except the negative control turned from blue to purple at 3.5 h. At 4.5 h, the wells other than the negative control were pink, while the negative control remained blue. This is because MRSA continued to grow and carry out its vital activities in the wells containing 1–2–4–8 μg/mL cef. As a result, the medium became acidic, and the blue anthocyanins turned pink. The colorimetric response is driven by the production of aOVCs by the bacteria, which triggers a structural change in the anthocyanin indicator from a quinonoidal base to a flavylium cation. Figure S1 in the Supporting Information illustrates the detailed chemical mechanism and protonation equilibrium. In Figure 2B, a light purple color was observed only in the well containing 1 μg/mL cef and the positive control at 3.5 h. At 4 h, the well containing 1 μg/mL cef and the positive control turned completely pink from blue. The well containing 2 μg/mL cef appeared light purple, the well containing 4 μg/mL cef appeared dark purple, and the other wells remained blue. After 6 h of incubation, the well containing 2 μg/mL cef turned pink. The well containing 4 μg/mL cef remained dark purple. This is because MSSA continued to grow and carry out its vital activities in wells containing 1 μg/mL and 2 μg/mL cef. The well containing 4 μg/mL cef remained a dark purple-blue color because it was the methicillin resistance threshold. There was no color change in the well containing 8 μg/mL cef. MSSA continued its vital activities at cef -sensitive doses (1 μg/mL and 2 μg/mL), releasing acidic components and causing the anthocyanin to protonate and shift to a pink color. At other doses, vital activities ceased due to inhibition, the environment did not acidify, and no visually discernible color differences were observed. Control experiments confirmed that the mineral oil overlay and antibiotics did not cause nonspecific pH changes, as the sterile blank wells showed no color change (Figure S2).

**Figure 2:**
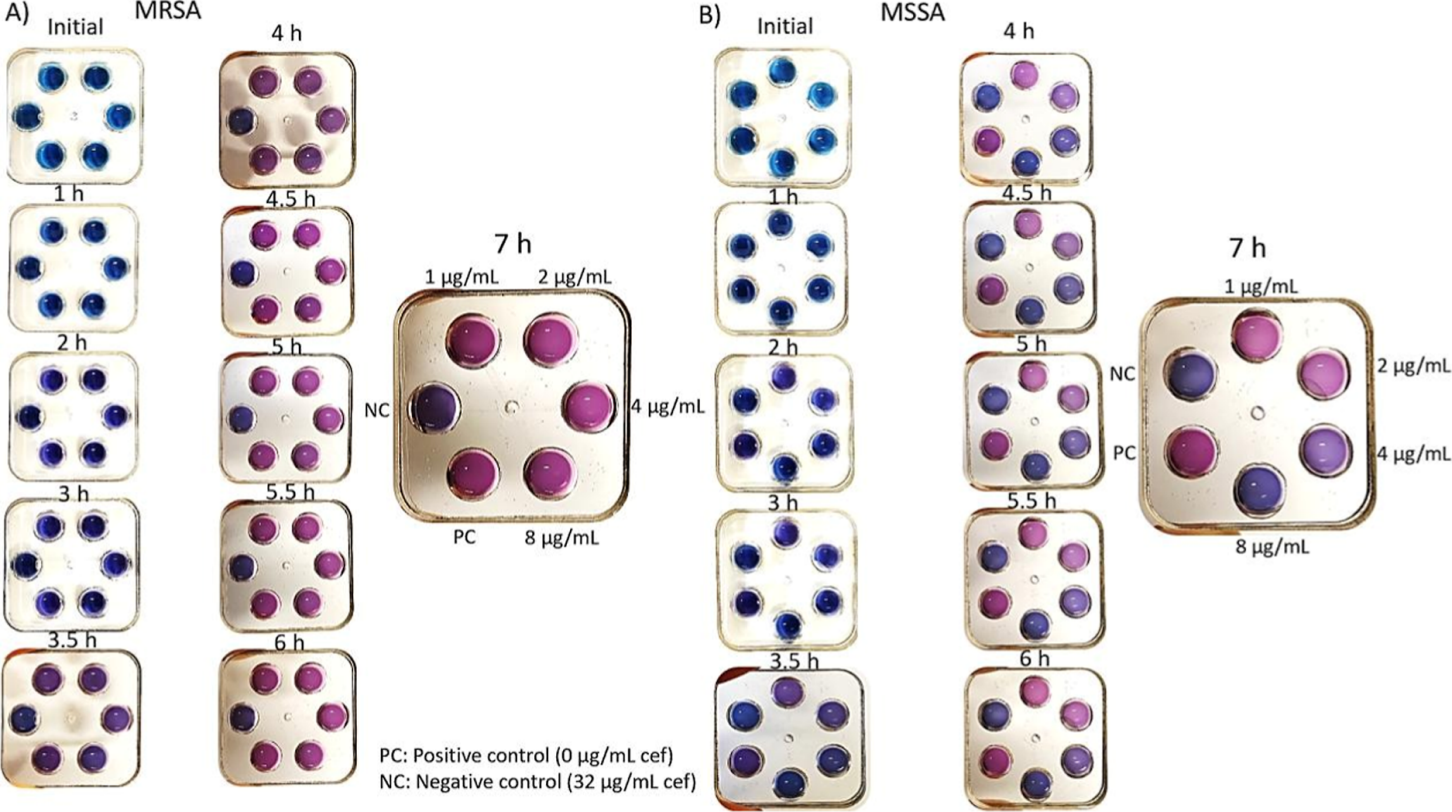
Time-dependent color change observed in the presence of MRSA in 6-well microfluidic chips. (A) MRSA analysis in microfluidic chips (B) MSSA analysis microfluidic chips.

In Figure 3, 5 μL of cef solution at concentrations of 1–2–4–8–16 μg/mL was added to the wells of 12-well microfluidic chips and dried. All antibiotic doses were prepared in duplicate. 1200 μL of anthocyanin test solution (pH 8, 1:4 anthocyanin ratio) was mixed with 1200 μL of 3 McFarland bacterial solution, and a total of 2400 μL of anthocyanin test solution was loaded into the center inlet well. Time-dependent color changes were recorded. The negative control contained 32 μg/mL cef, and the positive control contained 0 μg/mL cef. MRSA was tested in Figure 3A, and MSSA was tested in Figure 3B. After 3.5 h of incubation, wells containing 1–2–4 μg/mL cef and the positive control showed a color change from blue to purple, while 8–16 μg/mL and the negative control remained blue. After 4.5 h of incubation, the wells containing 1–2–4–8 μg/mL cef and the positive control turned pink, while the well containing 16 μg/mL cef remained light purple and the negative control remained dark purple. After 6 h of incubation, all wells except the negative control turned pink. MRSA triggered the color change by releasing aOVCs in wells containing 1–2–4–8–16 μg/mL cef.

**Figure 3:**
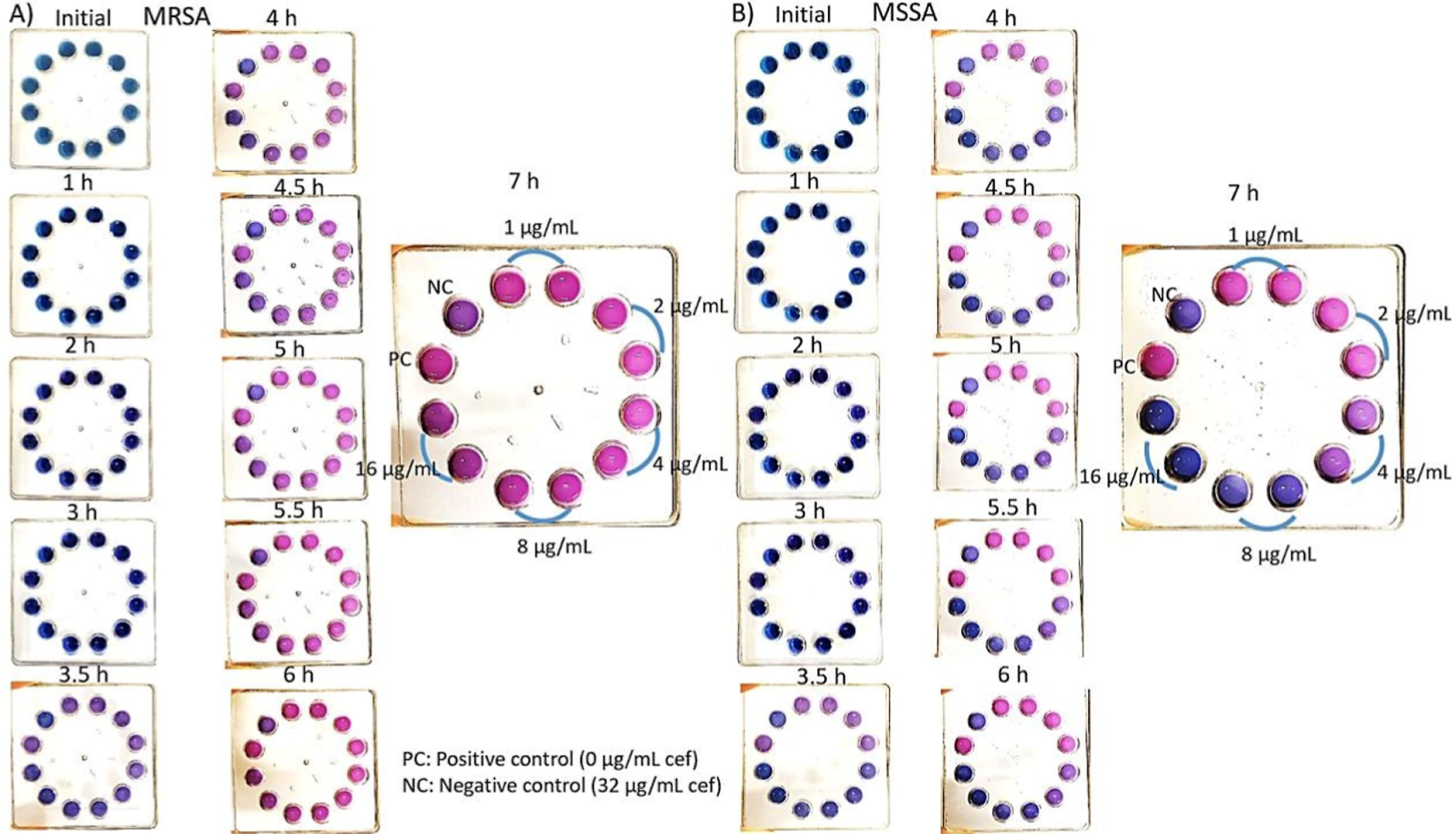
Time-dependent color change observed in the presence of MRSA in 12-well microfluidic chips. (A) MRSA analysis in microfluidic chips (B) MSSA analysis microfluidic chips.

In Figure 3B, MSSA was tested. After 3.5 h of incubation, wells containing 1–2 μg/mL cef and the positive control showed a color change from blue to purple, while wells containing 4–8–16 μg/mL and the negative control remained blue. After 4 h of incubation, the wells containing 1–2 μg/mL cef and the positive control turned pink, while the wells containing 4 μg/mL cef turned purple. Additionally, wells containing 8–16 μg/mL cef remained stable in blue-dark purple color. After 6 h of incubation, the condition remained stable as in the 4 h incubation. MSSA triggered the color change by releasing aOVCs in wells containing 1–2 μg/mL cef. Wells containing 4 μg/mL cef appeared in an intermediate color similar to Figure 3B, while MSSA did not continue its vital activities in wells containing 8–16 μg/mL cef. Direct pH measurements (Table S1) confirmed that growing MRSA caused significant metabolic acidification, while inhibited MSSA maintained stable pH, validating the metabolism-driven color change in wells containing 4–8 μg/mL cef.

Also, validation experiments comparing the microfluidic chip results with the gold-standard Broth Microdilution (BMD) method demonstrated 100% categorical agreement according to EUCAST guidelines, as detailed in Table S2 (Supporting Information).

It is known that the visual observation of colorimetric results may vary due to individual differences. To solve this problem, a semiquantitative analysis of the colorimetric results was performed using color image processing. For this purpose, the Red/Blue values of each well were calculated over time. An increase in this value is a numerical indicator that the well is moving away from blue and approaching pink. The R/B values obtained from MRSA and MSSA resistance analysis performed on 6-well and 12-well microfluidic chips are shown in Figure 4. Figure 4A,B show the results of MRSA and MSSA analysis on the 12-well microfluidic chip, while Figure 4C,D show the results of MRSA and MSSA analysis on the 6-well microfluidic chip.

**Figure 4:**
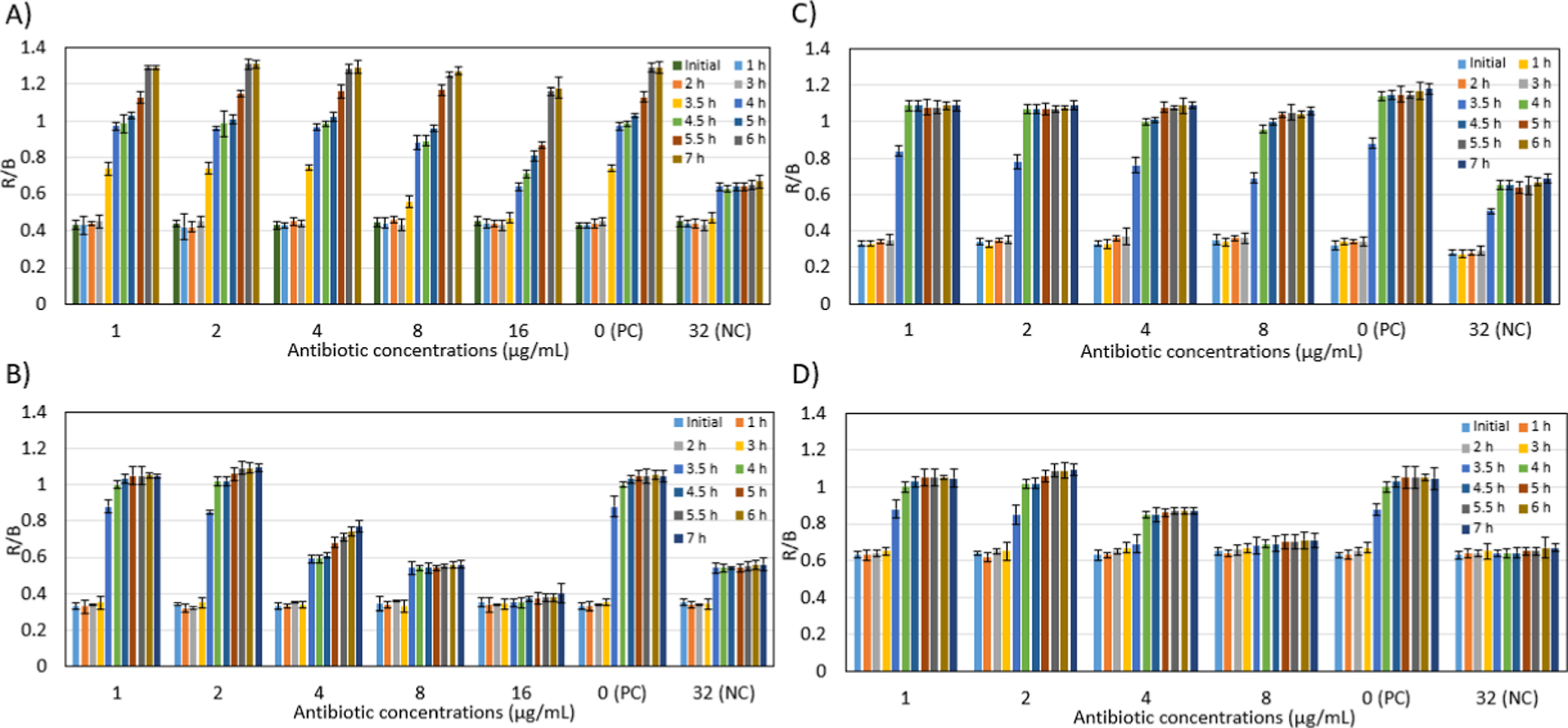
Time-dependent Red/Blue analysis in the presence of MRSA and MSSA in microfluidic chips. (A) MRSA analysis in 12-well microfluidic chips (B) MSSA analysis in 12-well microfluidic chips (C) MRSA analysis in 6-well microfluidic chips (D) MSSA analysis in 6-well microfluidic chips. Error bars represent the Standard Deviation (SD) of three independent experiments (*n* = 3).

Statistical framework and data presentation throughout this study, semiquantitative analysis of the colorimetric response was performed based on rigorous replication to ensure biological validity. Unless otherwise stated, all data points represent the mean values derived from three independent biological replicates (*n* = 3) performed on different days. Due to the miniaturized design of the POC device, each antibiotic concentration was tested in a single well on a 6-well microfluidic chip, and in two wells on a 12-well microfluidic chip. Consequently, the reported variability (Mean ± SD) and error bars in all figures reflect the interchip reproducibility across these independent biological replicates. Additionally, the RSD was calculated to quantify the consistency of the platform across different chip batches (Tables S3–S6). Statistical discrimination was determined using the 3σ decision rule as detailed in the Experimental Section.

In the 12-well microfluidic chip containing MRSA, the R/B value was 0.745 ± 0.017 in the well containing 4 μg/mL cef after 3.5 h of incubation, while it was 0.760 ± 0.042 in the 6-well microfluidic chip. After 4 h of incubation in the 12-well microfluidic chip, the R/B value was 0.966 ± 0.019 in the well containing 4 μg/mL cef, while it was 1.000 ± 0.016 in the 6-well microfluidic chip. After 3.5 h of incubation in the 12-well microfluidic chip, the R/B value in the well containing 8 μg/mL cef was 0.56 ± 0.032, while it was 0.690 ± 0.029 in the 6-well microfluidic chip. After 4 h of incubation in the 12- well microfluidic chip, the R/B value in the well containing 8 μg/mL cef was 0.88 ± 0.04, while it was 0.96 ± 0.02 in the 6-well microfluidic chip.

After 3.5 h of incubation in a 12-well microfluidic chip containing MSSA, the R/B value was 0.59 ± 0.021 in the well containing 4 μg/mL cef, while it was 0.69 ± 0.049 in the 6-well microfluidic chip. After 4 h of incubation in the 12-well microfluidic chip, the R/B value in the well containing 4 μg/mL cef was 0.59 ± 0.024, while it was 0.85 ± 0.016 in the 6-well microfluidic chip. After 3.5 h of incubation in the 12-well microfluidic chip, the R/B value was 0.54 ± 0.035 in the well containing 8 μg/mL cef, while it was 0.68 ± 0.047 in the 6-well microfluidic chip. After 4 h of incubation in the 12- well microfluidic chip, the R/B value was 0.54 ± 0.015 in the well containing 8 μg/mL cef, while it was 0.69 ± 0.02 in the 6-well microfluidic chip.

When evaluating which chip design yields effective results, the R/B difference between MRSA and MSSA is 0.155 in wells containing 4 μg/mL cef after 3.5 h of incubation in a 12- well microfluidic chip, while it is 0.100 in a 6-well microfluidic chip. After 4 h of incubation, the R/B difference between MRSA and MSSA was 0.376 in wells containing 4 μg/mL cef in the 12-well microfluidic chip, while it was 0.15 in the 6-well microfluidic chip. As a result, the R/B value difference between MRSA and MSSA after 4 h of incubation is higher in the 12-well microfluidic chip. The MRSA strain exhibited a significantly higher R/B ratio compared to the susceptible strain (Mean ± SD, *n* = 3). These color changes, which are difficult to distinguish visually, can be clearly calculated in the mobile application using the R/B difference.

The Delta E values obtained from MRSA and MSSA resistance analysis performed on 6-well and 12-well microfluidic chips are shown in Figure 5. Figure 5A,B show the results of MRSA and MSSA analysis on the 12-well microfluidic chip, while Figure 5C,D show the results of MRSA and MSSA analysis on the 6-well microfluidic chip. After 3.5 h of incubation in a 12-well microfluidic chip containing MRSA, the Delta E value was 36.2 ± 1.1 in the well containing 4 μg/mL cef, while it was 36.2 ± 1.3 in the 6-well microfluidic chip. After 4 h of incubation in a 12-well microfluidic chip, the Delta E value was 49.2 ± 1.2 in the well containing 4 μg/mL cef, while it was 49.2 ± 2.1 in the 6-well microfluidic chip. After 3.5 h of incubation in the 12-well microfluidic chip, the Delta E value in the well containing 8 μg/mL cef was 32.96 ± 1.3, while it was 23.2 ± 1.6 in the 6-well microfluidic chip. After 4 h of incubation in the 12-well microfluidic chip, the Delta E value in the well containing 8 μg/mL cef was 40.9 ± 1.8, while it was 35.09 ± 1.5 in the 6-well microfluidic chip.

**Figure 5:**
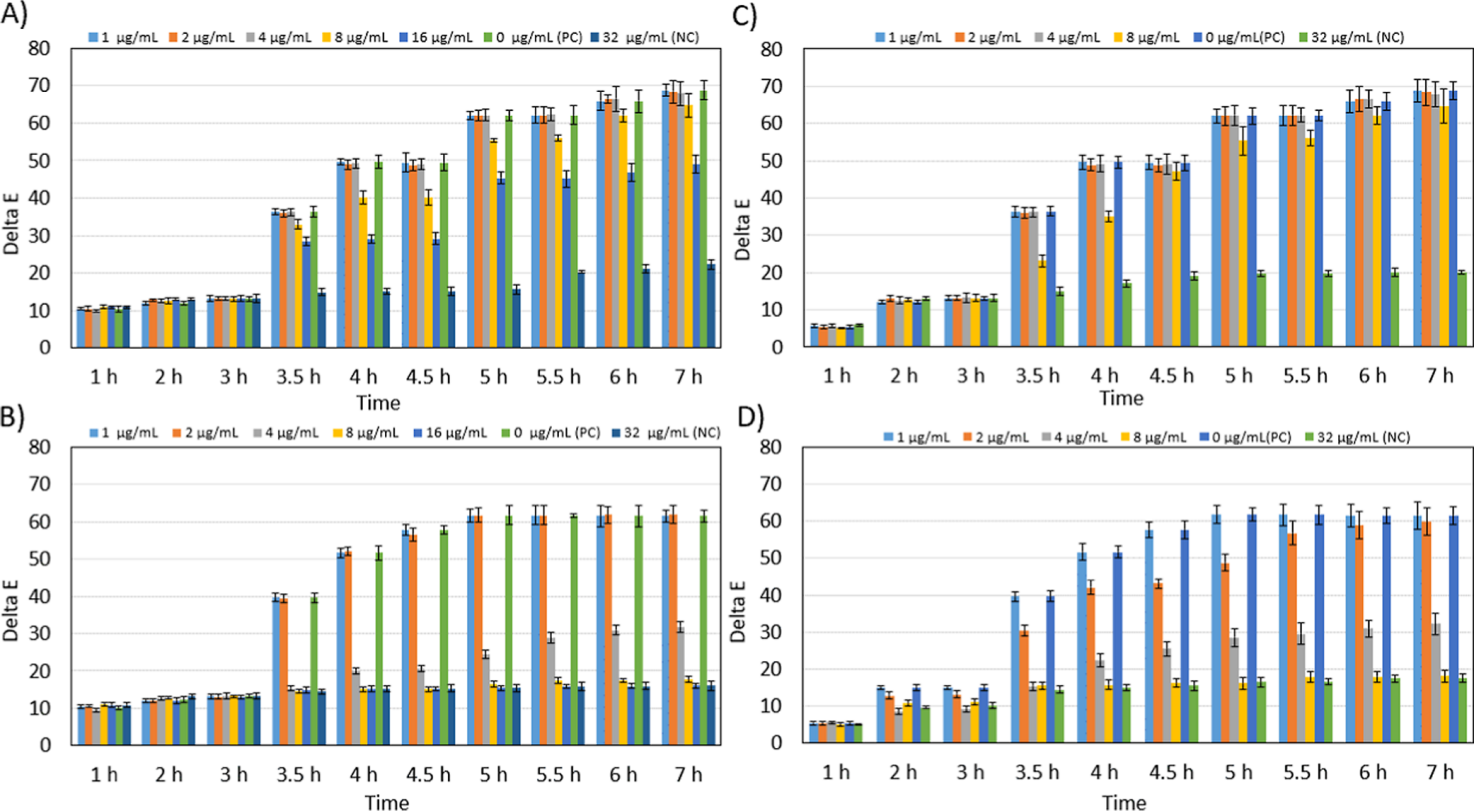
Time-dependent Delta E analysis in the presence of MRSA and MSSA in microfluidic chips. (A) MRSA analysis in 12-well microfluidic chips (B) MSSA analysis in 12- well microfluidic chips (C) MRSA analysis in 6-well microfluidic chips (D) MSSA analysis in 6-well microfluidic chips. Error bars represent the Standard Deviation (SD) of three independent experiments (*n* = 3).

After 3.5 h of incubation in the 12-well microfluidic chip containing MSSA, the Delta E value in the well containing 4 μg/mL cef was 15.36 ± 0.6, while it was 15.36 ± 1.2 in the 6-well microfluidic chip. After 4 h of incubation in the 12-well microfluidic chip, the Delta E value in the well containing 4 μg/mL cef was 20.1 ± 0.8, while it was 22.3 ± 1.7 in the 6-well microfluidic chip. After 3.5 h of incubation in the 12-well microfluidic chip, the Delta E value was 14.5 ± 0.5 in the well containing 8 μg/mL cef, while it was 15.5 ± 1.1 in the 6-well microfluidic chip. After 4 h of incubation in the 12-well microfluidic chip, the Delta E value in the well containing 8 μg/mL cef was 15.1 ± 0.6, while it was 15.8 ± 1.4 in the 6-well microfluidic chip.

When evaluating which chip design yields effective results, the Delta E value in the 12-well microfluidic chip containing MRSA in the well containing 8 μg/mL cef is much higher than the value in the 6-well microfluidic chip. Thus, it is clearly determined that the sample is MRSA. In the 12-well microfluidic chip containing MSSA, the Delta E value in the well containing 4 μg/mL cef is lower than the value in the 6-well microfluidic chip. The low color difference indicates that the color is close to the initial color and is inhibited due to its sensitivity. When both results are evaluated, it is considered that the 12-well microfluidic chip provides more effective results in resistance analysis. The performance comparison between the two microfluidic chip geometries highlighted the benefit of technical replication. Although the chemical mechanism is identical, the 12-well design includes duplicate wells for each antibiotic concentration, whereas the 6-well design uses a single well. By averaging the signals from these duplicate wells, the 12-well platform effectively mitigates random experimental variations (such as minor optical heterogeneities), leading to more consistent and reliable readouts than the single-point measurements of the 6-well format.

To evaluate the robust functionality of the microfluidic chip, basic performance parameters including detection range, sensitivity, and reliability were characterized. The chip was designed to cover the clinically relevant concentration range for cef. The tested antibiotic concentrations ranged from 1 μg/mL to 16 μg/mL, allowing for the precise determination of phenotypic resistance profiles consistent with EUCAST breakpoints.^26^ Representative statistical analysis results for both 6-well and 12-well microfluidic chips at the optimal readout time (4th h) are summarized in Tables 1 and 2, respectively. As demonstrated, the RSD values remained consistently below 5% at this critical decision point. Furthermore, the comprehensive data set covering all monitored time intervals (provided in the Supporting Information, Tables S3–S6) confirms that the method exhibits high precision, with RSD values remaining below 10% across all experimental conditions. As detailed in the statistical analysis section, the sensitivity of the system relies on the discrimination threshold (*T* = μ_susceptible_ + 3σ_susceptible_). The system successfully distinguished MRSA growth from MSSA inhibition even at the lowest meaningful color change, ensuring no false-negative results within the tested conditions.

**Table 1:** Statistical Analysis Results of the 6-Well Microfluidic Chips at the Detection Time (4th h)

strain	Cef concentration (μg/mL)	mean R/B	SD	RSD (%)
MRSA	0 (PC)	1.14	0.025	2.20
	1	1.09	0.028	2.57
	2	1.07	0.024	2.24
	4	1.00	0.016	1.60
	8	0.96	0.02	2.08
	32 (NC)	0.65	0.024	3.70
MSSA	0 (PC)	1.00	0.025	2.50
	1	1.00	0.028	2.80
	2	1.02	0.024	2.35
	4	0.85	0.016	1.88
	8	0.69	0.020	2.90
	32 (NC)	0.64	0.024	3.75

**Table 2:** Statistical Analysis Results of the 12-Well Microfluidic Chips at the Detection Time (4th h)

Strain	Cef concentration (μg/mL)	Mean R/B	SD	RSD (%)
MRSA	0 (PC)	0.97	0.019	1.96
	1	0.97	0.019	1.96
	2	0.96	0.009	0.94
	4	0.97	0.019	1.97
	8	0.88	0.039	4.43
	16	0.64	0.023	3.60
	32 (NC)	0.64	0.019	2.97
MSSA	0 (PC)	1.00	0.013	1.30
	1	1.00	0.020	2.00
	2	1.02	0.025	2.45
	4	0.59	0.024	4.07
	8	0.54	0.015	2.78
	16	0.35	0.015	4.28
	32 (NC)	0.54	0.020	3.70

A 3D-printed smartphone platform was developed and a mobile application compatible with microfluidic chips was designed. As shown in Figure 6, it was used to analyze digital images using R/B and ED calculations. The smartphone platform is compatible with both 6-well and 12-well microfluidic chips (Figure 6A). The mobile application is also designed to analyze the color of each well separately (Figure 6B).

**Figure 6:**
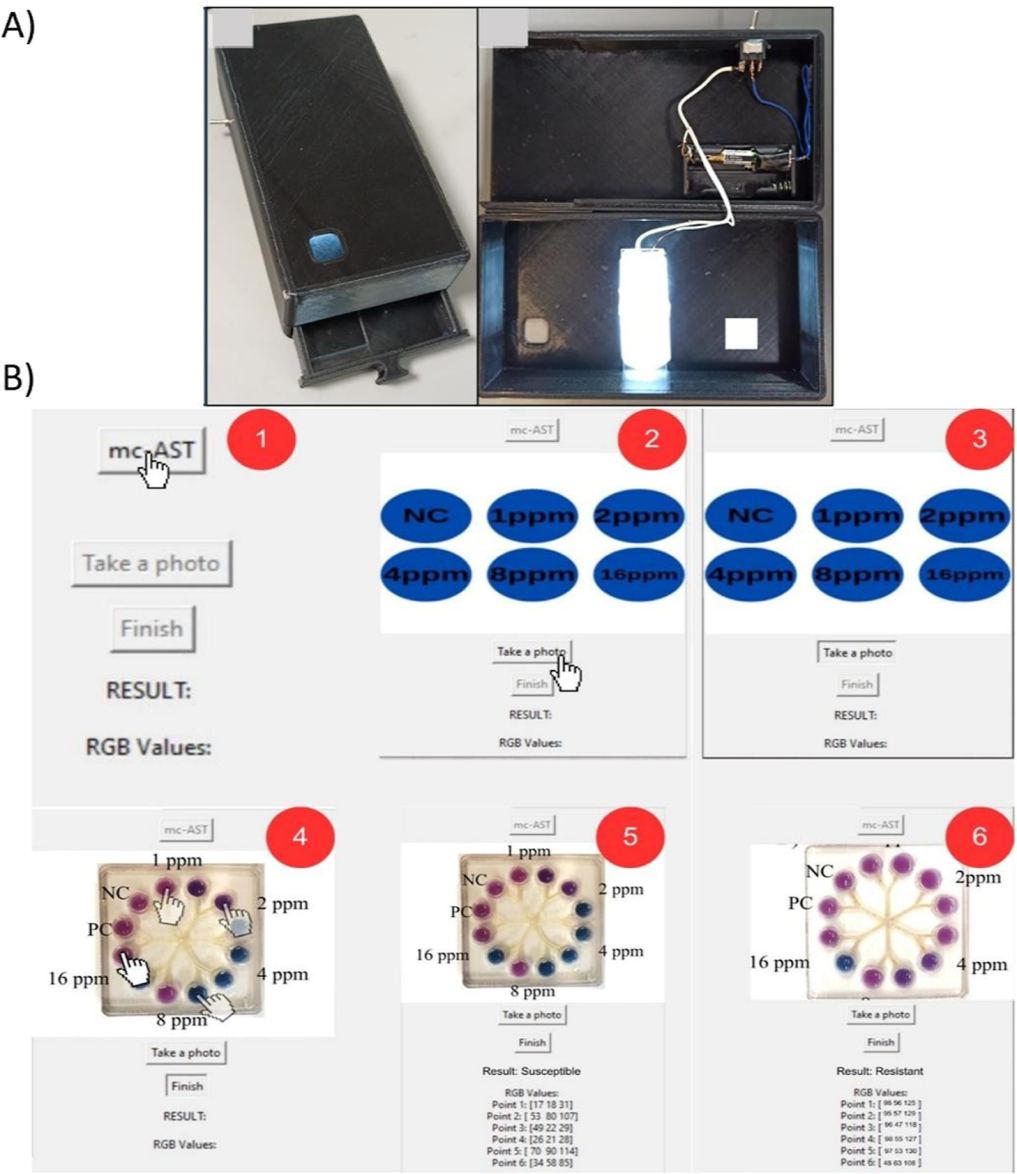
Images of smartphone platform and mobile application interfaces for analysis (A) Images of the smartphone platform from different viewpoints (B) User-friendly interfaces of smartphone applications.

To validate the analytical accuracy of this application, the calculated R/B ratios were compared against measurements obtained from standard image analysis software (ImageJ). As illustrated in Figure S3 (Supporting Information), a strong linear correlation (*R* ^2^ > 0.98) was verified, confirming that the smartphone-based quantification is reliable and comparable to benchtop benchmarks.

While the fundamental sensing chemistry utilizes the optimized anthocyanin parameters we previously reported,^20,22,23^ the current study represents a significant leap in system integration and engineering. Unlike our previous macro-scale or single-analyte investigations, this work introduces a monolithic microfluidic architecture capable of multiplexed analysis. As detailed in Table S7 (Supporting Information), key innovations include the development of a pump-free central distribution network, the integration of preloaded antibiotics for a “ready-to-use” workflow, and the validation of interchip reproducibility in a miniaturized format. Thus, this platform translates a proven chemical principle into a standalone, field-deployable POC product.

A comprehensive comparison of the developed microfluidic chip with recently reported state-of-the-art AST platforms is provided in Table S8 (Supporting Information). While offering comparable diagnostic accuracy, the proposed platform distinguishes itself by delivering results within 4 h (rapid time-to-result) without requiring bulky instrumentation, highlighting its advantage as a cost-effective and operationally simple solution for resource-limited settings.

## 4 Limitations of the Study

The main limitation of this study is that it focuses on a defined set of standard reference strains (*S. aureus* ATCC 43300 and ATCC 25923), rather than a diverse panel of clinical isolates. While these strains provide a controlled approach to demonstrating device functionality, they do not fully capture the biological heterogeneity encountered in clinical settings. Consequently, it was not possible to establish statistical diagnostic performance metrics such as clinical sensitivity and specificity in this phase. This study serves as proof-of-concept, demonstrating the analytical feasibility of the smartphone-based colorimetric assay. Future studies will focus on validating the device against a larger library of patient-derived isolates, comparing the results with those of the reference BMD, and determining comprehensive diagnostic accuracy.

Regarding potential interference from nontarget microorganisms, it is important to note that this platform is designed as a downstream AST tool following standard bacterial isolation. Similar to gold-standard BMD or commercial automated systems, the assay requires an isolated colony suspension to ensure that the metabolic signal is derived exclusively from the target pathogen. Therefore, interference from mixed cultures is mitigated by the standard clinical workflow, which mandates the isolation of pure cultures prior to susceptibility testing.

## 5 Conclusion

We developed colorimetric phenotypic antimicrobial susceptibility tests using microfluidic chip technology to detect MRSA. We also designed a mobile application and smartphone platform to analyze these test results. In this study, methicillin resistance was detected more quickly than with other phenotypic colorimetric tests, and the resistance profile against several different doses was revealed simultaneously. This reduced the workload and minimized the need for expensive equipment. In the presence of cefoxitin, MRSA continued to grow and released aOVCs into the environment, while MSSA was inhibited. The aOVCs produced colorimetric results by acidifying the wells. We demonstrated that color changes could be distinguished both by the naked eye and through color image processing techniques. As a proof-of-concept study utilizing standard reference strains, these findings demonstrate the feasibility of the proposed platform. Thus, this innovative solution offers a promising potential tool to combat antibiotic resistance, pending further validation with clinical isolates.

## Supplementary Material



## Notes

Schematic representation of the pH-dependent structural transformation of anthocyanins (Figure S1); assay specificity and negative controls (Figure S2); direct pH measurements of microfluidic wells (Table S1); comparison of MIC results obtained from the microfluidic chip and the standard reference method (Table S2); raw colorimetric data (Tables S3–S6); validation of the smartphone-based image analysis algorithm (Figure S3); operational comparison between the proposed microfluidic chip and prior manual assays (Table S7); and comparison of the operational characteristics of the developed smartphone-based microfluidic chip with other recently reported antimicrobial susceptible testing (AST) methods from the literature (Table S8) (PDF)
