# Supplementary material for: Colorimetric Ready-to-Use Microfluidic Chips for Rapid Antibiotic Susceptibility Testing of Methicillin-Resistant Staphylococcus aureus by a Smartphone-Based Analysis
Source: ACS Omega. 2026 Mar 9;11(11):18144–54. doi: 10.1021/acsomega.5c13266 (PMC13019210; doi:10.1021/acsomega.5c13266)
Supplement: Supplementary file 1 [file ao5c13266_si_001.pdf]

SUPPORTING INFORMATION

Colorimetric Ready-to-Use Microfluidic Chips for Rapid Antibiotic Susceptibility Testing of Methicillin-resistant *Staphylococcus aureus* by a Smartphone-based Analysis

Cagla Celik Yoldas[a], Nilay Ildiz[b], Naim Yagiz Demir[c,d], Memed Duman[d], Erhan Yoldas[e], Sadik Kucukgunay[f], Kadir Erol[g], Ismail Ocsoy[h]*

[a]Department of Analytical Chemistry, Faculty of Pharmacy, Harran University, 63200, Sanliurfa, Türkiye

[b]Medical Imaging Department, Vocational School of Health Services, Bandirma Onyedi Eylul University, 10200, Balikesir, Türkiye

[c]Department of Oceanography, Institute of Marine Sciences, Middle East Technical University, 33731, Mersin, Türkiye

[d]Nanotechnology and Nanomedicine Division, Institute of Science, Hacettepe University, 06100, Ankara, Türkiye

[e]Department of Electrical and Electronics, Faculty of Engineering, Harran University, 63200, Sanliurfa, Türkiye

[f]Department of Medical Pharmacology, Faculty of Medicine, Kirsehir Ahi Evran University, 40100, Kirsehir, Türkiye

[g]Department of Medical Services and Techniques, Vocational School of Health Services, Hitit University, 19030, Corum, Türkiye

[h]Department of Analytical Chemistry, Faculty of Pharmacy, Erciyes University, 38039, Kayseri, Türkiye

*E-mail: caglacelik@harran.edu.tr

1. Experimental Section

Following the loading and drying of antibiotic agents, the microfluidic chips were sealed with an adhesive film and stored at 4°C in a container with silica gel desiccants to prevent moisture absorption. Under these conditions, the pre-loaded chips demonstrated stability for at least 4 weeks, showing no significant deviation in Minimum Inhibitory Concentration (MIC) determination performance compared to freshly prepared references. To verify fabrication reproducibility, the Relative Standard Deviation (RSD) of the colorimetric response across independent chip batches was calculated to be less than 10%, indicating high lot-to-lot consistency (Table S3-S6).

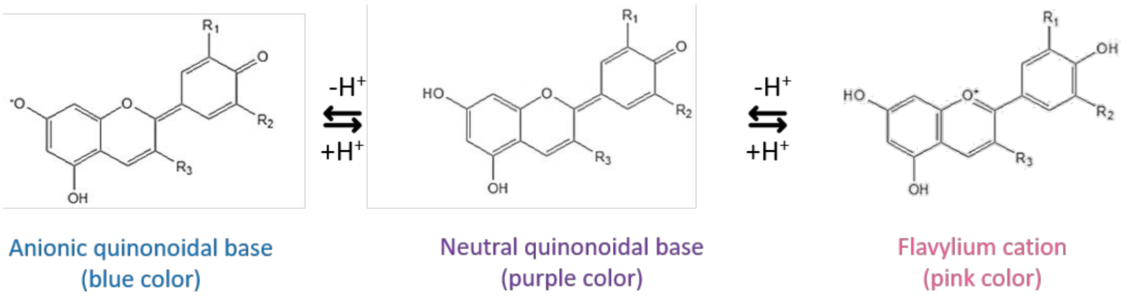

Figure S1. Schematic representation of the pH-dependent structural transformation of anthocyanins [1,2].

Under alkaline conditions (pH~8), the molecule exists in an anionic quinonoid base form (shown in blue), whereas under neutral conditions (pH~7), it exists in a neutral quinonoid base form (shown in purple) in Figure S1. With bacterial growth and aOVCs production (pH<5), protonation leads to a shift in equilibrium towards the flavylium cation form (pink color). This mechanism enables resistant bacteria to be visually distinguished from susceptible bacteria.

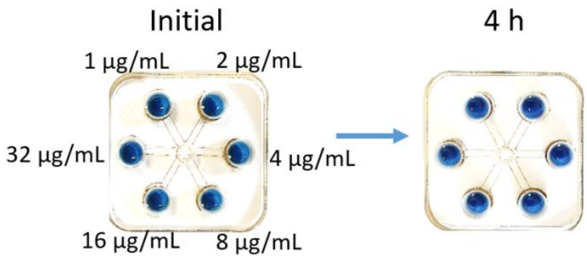

Figure S2. Assay specificity and negative controls.

Representative images of sterile blank wells containing the test medium, antibiotic (1–32 µg/mL), anthocyanin indicator, and mineral oil overlay without bacterial inoculation. The wells maintained their initial blue color throughout the incubation period. This confirms that the

mineral oil overlay and antibiotic agents did not induce any non-specific acidification or optical interference in the absence of bacterial metabolism.

Table S1. Direct pH measurements of microfluidic wells.

**MRSA**	Initial	4 h	**MSSA**	Initial	4 h
1 µg/mL	8.03	2.6	1 µg/mL	8.03	2.6
2 µg/mL	8.03	2.6	2 µg/mL	8.03	2.6
4 µg/mL	8.03	2.7	4 µg/mL	8.03	7.1
8 µg/mL	8.03	3.8	8 µg/mL	8.03	7.8
16 µg/mL	8.03	4.3	16 µg/mL	8.03	8.01
32 µg/mL	8.03	7.6	32 µg/mL	8.03	8.01

Final pH values of the reaction medium in microfluidic wells measured after 4 h of incubation for MRSA and MSSA strains. Measurements were performed using a micro-pH probe. Note that MRSA growth in the presence of cef (1–32 µg/mL) resulted in a significant pH change, leading to the observed color change from blue to pink. In contrast, inhibited MSSA wells maintained their initial basic pH.

Table S2. Comparison of MIC results obtained from the microfluidic chip and the standard reference method.

**Bacterial Strain**	**Phenotype**	**Reference Method (BMD) Result**	**EUCAST Breakpoint Criteria**	**Microfluidic Chip Result**	**Classification (EUCAST/CLSI)**	**Agreement**
S. aureus ATCC 43300	MRSA	MIC ≥ 16 µg/mL	R > 4 µg/mL	≥ 16 µg/mL	Resistant	100%
S. aureus ATCC 25923	MSSA	MIC 1-4 µg/mL	S ≤ 4 µg/mL	4 µg/mL	Susceptible	100%

To validate the diagnostic accuracy of the microfluidic chip platform, the MIC values determined by the microfluidic chip were compared with those obtained using the standard Broth Microdilution (BMD) method. The interpretation of resistance was based on EUCAST 2026 guidelines (Resistance breakpoint for *S. aureus* > 4 mg/L cef) [3]. As summarized in Table S6, the microfluidic platform correctly classified both reference strains. The MRSA strain (ATCC 43300) showed growth in all wells (MIC ≥ 16 µg/mL), consistent with the reference BMD result. Conversely, the MSSA strain (ATCC 25923) was inhibited at all concentrations (MIC ≤ 4 µg/mL), aligning with its susceptible profile. This resulted in 100% categorical agreement between the proposed chip and the gold-standard method.

2. Results and Discussion

Table S3. Detailed statistical data (Mean, SD, and RSD %) of colorimetric response (R/B) over time for MRSA and MSSA strains in 12-well microfluidic chips.

**R/B**	1 µg/mL			2 µg/mL			4 µg/mL			8 µg/mL			16 µg/mL			0 µg/mL (PC)			32 µg/mL (NC)		
**MRSA**	Mean	SD	RSD (%)	Mean	SD	RSD (%)	Mean	SD	RSD (%)	Mean	SD	RSD (%)	Mean	SD	RSD (%)	Mean	SD	RSD (%)	Mean	SD	RSD (%)
Initial	0.43	0.024	5.581395	0.44	0.016	3.636364	0.43	0.02	4.651163	0.445	0.025	5.617978	0.452	0.024	5.309735	0.43	0.015	3.488372	0.452	0.028	6.19469
1 h	0.43	0.042	9.767442	0.42	0.07	16.66667	0.43	0.013	3.023256	0.44	0.028	6.363636	0.44	0.024	5.454545	0.43	0.016	3.72093	0.44	0.02	4.545455
2 h	0.44	0.013	2.954545	0.42	0.028	6.666667	0.45	0.024	5.333333	0.46	0.016	3.478261	0.44	0.02	4.545455	0.44	0.025	5.681818	0.44	0.024	5.454545
3 h	0.45	0.035	7.777778	0.45	0.03	6.666667	0.44	0.017	3.863636	0.43	0.032	7.44186	0.43	0.028	6.511628	0.45	0.018	4	0.43	0.029	6.744186
3.5 h	0.739	0.035	4.73613	0.741	0.03	4.048583	0.745	0.017	2.281879	0.56	0.032	5.714286	0.47	0.028	5.957447	0.739	0.018	2.435724	0.47	0.029	6.170213
4 h	0.97	0.019	1.958763	0.96	0.009	0.9375	0.966	0.019	1.966874	0.88	0.039	4.431818	0.64	0.023	3.59375	0.97	0.019	1.958763	0.64	0.019	2.96875
4.5 h	0.985	0.05	5.076142	0.986	0.07	7.099391	0.985	0.013	1.319797	0.89	0.028	3.146067	0.71	0.024	3.380282	0.985	0.016	1.624365	0.63	0.02	3.174603
5 h	1.03	0.02	1.941748	1.01	0.025	2.475248	1.02	0.024	2.352941	0.96	0.015	1.5625	0.81	0.028	3.45679	1.03	0.013	1.262136	0.64	0.02	3.125
5.5 h	1.13	0.03	2.654867	1.15	0.017	1.478261	1.16	0.032	2.758621	1.17	0.028	2.393162	0.87	0.018	2.068966	1.13	0.029	2.566372	0.64	0.021	3.28125
6 h	1.29	0.013	1.007752	1.31	0.028	2.137405	1.28	0.024	1.875	1.25	0.016	1.28	1.16	0.02	1.724138	1.29	0.025	1.937984	0.65	0.024	3.692308
7 h	1.29	0.012	0.930233	1.31	0.021	1.603053	1.29	0.035	2.713178	1.27	0.02	1.574803	1.18	0.054	4.576271	1.29	0.031	2.403101	0.67	0.035	5.223881

**R/B**	1 µg/mL			2 µg/mL			4 µg/mL			8 µg/mL			16 µg/mL			0 µg/mL (PC)			32 µg/mL (NC)		
**MSSA**	Mean	SD	RSD (%)	Mean	SD	RSD (%)	Mean	SD	RSD (%)	Mean	SD	RSD (%)	Mean	SD	RSD (%)	Mean	SD	RSD (%)	Mean	SD	RSD (%)
Initial	0.33	0.019	5.757576	0.34	0.009	2.647059	0.33	0.019	5.757576	0.345	0.039	11.30435	0.352	0.023	6.534091	0.33	0.019	5.757576	0.352	0.019	5.397727
1 h	0.33	0.032	9.69697	0.32	0.019	5.9375	0.33	0.009	2.727273	0.34	0.019	5.588235	0.34	0.039	11.47059	0.33	0.023	6.969697	0.34	0.019	5.588235
2 h	0.34	0.004	1.176471	0.32	0.005	1.5625	0.35	0.005	1.428571	0.36	0.005	1.388889	0.34	0.004	1.176471	0.34	0.004	1.176471	0.34	0.004	1.176471
3 h	0.35	0.035	10	0.35	0.03	8.571429	0.34	0.017	5	0.33	0.032	9.69697	0.343	0.028	8.163265	0.35	0.018	5.142857	0.343	0.029	8.45481
3.5 h	0.88	0.0338	3.840909	0.85	0.012	1.411765	0.59	0.021	3.559322	0.54	0.035	6.481481	0.35	0.020	5.714286	0.88	0.054	6.136364	0.54	0.031	5.740741
4 h	1	0.02	2	1.02	0.025	2.45098	0.59	0.024	4.067797	0.54	0.015	2.777778	0.35	0.015	4.285714	1	0.013	1.3	0.54	0.02	3.703704
4.5 h	1.03	0.025	2.427184	1.02	0.024	2.352941	0.61	0.015	2.459016	0.54	0.028	5.185185	0.37	0.013	3.513514	1.03	0.02	1.941748	0.54	0.01	1.851852
5 h	1.05	0.048	4.571429	1.06	0.035	3.301887	0.68	0.03	4.411765	0.54	0.017	3.148148	0.375	0.032	8.533333	1.05	0.028	2.666667	0.54	0.018	3.333333
5.5 h	1.05	0.0479	4.561905	1.09	0.035	3.211009	0.71	0.019	2.676056	0.55	0.009	1.636364	0.378	0.019	5.026455	1.05	0.039	3.714286	0.55	0.023	4.181818
6 h	1.052	0.013	1.235741	1.09	0.028	2.568807	0.74	0.024	3.243243	0.559	0.016	2.862254	0.379	0.02	5.277045	1.052	0.025	2.376426	0.559	0.024	4.293381
7 h	1.048	0.012	1.145038	1.095	0.021	1.917808	0.77	0.035	4.545455	0.559	0.02	3.577818	0.401	0.054	13.46633	1.048	0.031	2.958015	0.559	0.035	6.261181

Table S4. Detailed statistical data (Mean, SD, and RSD %) of colorimetric response (R/B) over time for MRSA and MSSA strains in 6-well microfluidic chips.

**R/B**	1 µg/mL			2 µg/mL			4 µg/mL			8 µg/mL			0 µg/mL (PC)			32 µg/mL (NC)		
**MRSA**	Mean	SD	RSD (%)	Mean	SD	RSD (%)	Mean	SD	RSD (%)	Mean	SD	RSD (%)	Mean	SD	RSD (%)	Mean	SD	RSD (%)
Initial	0.33	0.015	4.545455	0.34	0.017	5	0.33	0.013	3.939394	0.35	0.028	8	0.32	0.024	7.5	0.28	0.016	5.714286
1 h	0.33	0.013	3.939394	0.32	0.028	8.75	0.33	0.024	7.272727	0.34	0.016	4.705882	0.34	0.02	5.882353	0.27	0.025	9.259259
2 h	0.34	0.013	3.823529	0.35	0.014	4	0.36	0.012	3.333333	0.36	0.012	3.333333	0.34	0.014	4.117647	0.28	0.014	5
3 h	0.35	0.0267	7.628571	0.35	0.021	6	0.37	0.049	13.24324	0.36	0.028	7.777778	0.34	0.0249	7.323529	0.29	0.026	8.965517
3.5 h	0.84	0.025	2.97619	0.78	0.039	5	0.76	0.042	5.526316	0.69	0.0289	4.188406	0.88	0.0277	3.147727	0.51	0.0135	2.647059
4 h	1.09	0.028	2.568807	1.07	0.024	2.242991	1	0.016	1.6	0.96	0.02	2.083333	1.14	0.025	2.192982	0.65	0.024	3.692308
4.5 h	1.09	0.028	2.568807	1.07	0.024	2.242991	1.01	0.016	1.584158	1	0.02	2	1.15	0.025	2.173913	0.65	0.024	3.692308
5 h	1.08	0.042	3.888889	1.07	0.0289	2.700935	1.08	0.0277	2.564815	1.04	0.0135	1.298077	1.15	0.043	3.73913	0.64	0.031	4.84375
5.5 h	1.08	0.0378	3.5	1.07	0.02	1.869159	1.08	0.01	0.925926	1.05	0.042	4	1.15	0.017	1.478261	0.65	0.0475	7.307692
6 h	1.09	0.02	1.834862	1.08	0.01	0.925926	1.09	0.042	3.853211	1.04	0.017	1.634615	1.17	0.0475	4.059829	0.67	0.02	2.985075
7 h	1.09	0.028	2.568807	1.09	0.024	2.201835	1.09	0.016	1.46789	1.06	0.02	1.886792	1.18	0.025	2.118644	0.69	0.024	3.478261

**R/B**	1 µg/mL			2 µg/mL			4 µg/mL			8 µg/mL			0 µg/mL (PC)			32 µg/mL (NC)		
**MSSA**	Mean	SD	RSD (%)	Mean	SD	RSD (%)	Mean	SD	RSD (%)	Mean	SD	RSD (%)	Mean	SD	RSD (%)	Mean	SD	RSD (%)
Initial	0.63	0.017	2.698413	0.64	0.013	2.03125	0.63	0.028	4.444444	0.65	0.024	3.692308	0.63	0.016	2.539683	0.63	0.02	3.174603
1 h	0.63	0.028	4.444444	0.62	0.024	3.870968	0.63	0.016	2.539683	0.64	0.02	3.125	0.63	0.025	3.968254	0.64	0.024	3.75
2 h	0.64	0.015	2.34375	0.65	0.017	2.615385	0.65	0.013	2	0.66	0.028	4.242424	0.65	0.024	3.692308	0.64	0.016	2.5
3 h	0.65	0.021	3.230769	0.65	0.049	7.538462	0.67	0.028	4.179104	0.67	0.0249	3.716418	0.67	0.026	3.880597	0.65	0.04	6.153846
3.5 h	0.88	0.048	5.454545	0.85	0.05	5.882353	0.69	0.049	7.101449	0.68	0.047	6.911765	0.88	0.032	3.636364	0.64	0.018	2.8125
4 h	1	0.028	2.8	1.02	0.024	2.352941	0.85	0.016	1.882353	0.69	0.02	2.898551	1	0.025	2.5	0.64	0.024	3.75
4.5 h	1.03	0.0279	2.708738	1.02	0.025	2.45098	0.85	0.039	4.588235	0.69	0.042	6.086957	1.03	0.0289	2.805825	0.64	0.0277	4.328125
5 h	1.05	0.047	4.47619	1.06	0.032	3.018868	0.86	0.018	2.093023	0.7	0.038	5.428571	1.05	0.059	5.619048	0.65	0.021	3.230769
5.5 h	1.05	0.047	4.47619	1.09	0.032	2.93578	0.87	0.018	2.068966	0.7	0.038	5.428571	1.05	0.059	5.619048	0.65	0.021	3.230769
6 h	1.052	0.01	0.95057	1.09	0.042	3.853211	0.87	0.017	1.954023	0.71	0.0475	6.690141	1.052	0.02	1.901141	0.67	0.054	8.059701
7 h	1.048	0.047	4.484733	1.095	0.032	2.922374	0.87	0.018	2.068966	0.71	0.038	5.352113	1.048	0.059	5.629771	0.67	0.021	3.134328

Precision values (RSD %) were generally found to be below 10%. Although slightly higher RSD values (>10%) were observed at certain concentrations, these are considered acceptable POC devices and biological assays involving bacterial growth, where inherent biological heterogeneity and minor variations in ambient lighting can influence the readout. Importantly, these variations did not affect the accuracy of the MIC breakpoint determination.

Table S5. Detailed statistical data (Mean, SD, and RSD %) of colorimetric response (Delta E) over time for MRSA and MSSA strains in 12-well microfluidic chips.

**Delta E**	1 µg/mL			2 µg/mL			4 µg/mL			8 µg/mL			16 µg/mL			0 µg/mL (PC)			32 µg/mL (NC)		
**MRSA**	Mean	SD	RSD (%)	Mean	SD	RSD (%)	Mean	SD	RSD (%)	Mean	SD	RSD (%)	Mean	SD	RSD (%)	Mean	SD	RSD (%)	Mean	SD	RSD (%)
1 h	10.5	0.2	1.904762	10.52	0.5	4.752852	9.87	0.4	4.052685	11.02	0.5	4.537205	10.89	0.3	2.754821	10.4	0.7	6.730769	10.89	0.4	3.673095
2 h	12.04	0.4	3.322259	12.89	0.3	2.327386	12.54	0.5	3.987241	12.7	0.8	6.299213	13.01	0.3	2.305919	12.04	0.4	3.322259	13.01	0.3	2.305919
3 h	13.15	0.8	6.08365	13.18	0.5	3.793627	13.25	0.6	4.528302	13.14	0.6	4.56621	13.21	0.7	5.299016	13.15	0.6	4.562738	13.21	1.1	8.327025
3.5 h	36.4	0.9	2.472527	35.9	1.02	2.841226	36.2	1.1	3.038674	32.96	1.3	3.944175	28.49	1.2	4.212004	36.4	1.4	3.846154	14.9	1.02	6.845638
4 h	49.6	0.7	1.41129	48.9	1.2	2.453988	49.2	1.2	2.439024	40.09	1.8	4.489898	29.06	1.2	4.129387	49.6	1.8	3.629032	15.2	0.8	5.263158
4.5 h	49.5	2.5	5.050505	48.8	1.42	2.909836	49.02	1.3	2.651979	40.1	2	4.987531	29.12	1.6	5.494505	49.5	2.2	4.444444	15.21	1.1	7.232084
5 h	61.98	1.2	1.936108	62.01	1.52	2.451218	62.16	1.7	2.734878	55.3	0.4	0.723327	45.3	1.52	3.355408	61.98	1.4	2.258793	15.64	1.2	7.672634
5.5 h	62.14	2.3	3.70132	62.18	2.1	3.377292	62.24	1.8	2.892031	56.03	0.9	1.606282	45.12	2.2	4.875887	62.14	2.5	4.023173	20.23	0.3	1.482946
6 h	65.9	2.5	3.793627	66.5	1.1	1.654135	66.46	3.3	4.965393	62.1	1.8	2.898551	46.74	2.3	4.920839	65.9	3	4.552352	21.23	1.1	5.181347
7 h	68.8	1.7	2.47093	68.4	3.1	4.532164	67.9	3.1	4.565538	64.8	3.1	4.783951	48.9	2.4	4.907975	68.8	2.5	3.633721	22.31	1.2	5.378754

**Delta E**	1 µg/mL			2 µg/mL			4 µg/mL			8 µg/mL			16 µg/mL			0 µg/mL (PC)			32 µg/mL (NC)		
**MSSA**	Mean	SD	RSD (%)	Mean	SD	RSD (%)	Mean	SD	RSD (%)	Mean	SD	RSD (%)	Mean	SD	RSD (%)	Mean	SD	RSD (%)	Mean	SD	RSD (%)
1 h	10.4	0.5	4.807692	10.5	0.3	2.857143	9.5	0.4	4.210526	11.1	0.5	4.504505	10.9	0.6	5.504587	10.1	0.5	4.950495	10.89	0.7	6.427916
2 h	12.1	0.5	4.132231	12.1	0.4	3.305785	12.7	0.6	4.724409	12.8	0.4	3.125	12.1	0.7	5.785124	12.3	0.7	5.691057	13.2	0.7	5.30303
3 h	13.2	0.6	4.545455	13.1	0.6	4.580153	13.3	0.8	6.015038	13.2	0.4	3.030303	13.1	0.5	3.816794	13.3	0.6	4.511278	13.2	0.8	6.060606
3.5 h	39.6	1.1	2.777778	39.4	1.2	3.045685	15.36	0.6	3.90625	14.5	0.5	3.448276	14.9	0.7	4.697987	39.6	1.2	3.030303	14.5	0.7	4.827586
4 h	51.7	1.2	2.321083	52.1	1.2	2.303263	20.1	0.8	3.9801	15.1	0.6	3.97351	15.2	0.7	4.605263	51.7	1.8	3.481625	15.2	0.9	5.921053
4.5 h	57.8	1.4	2.422145	56.7	1.8	3.174603	20.5	0.8	3.902439	15.1	0.7	4.635762	15.21	0.4	2.629849	57.8	1.1	1.903114	15.3	1	6.535948
5 h	61.79	1.8	2.913093	61.8	1.9	3.074434	24.5	1.1	4.489796	16.5	0.7	4.242424	15.4	0.5	3.246753	61.79	2.5	4.045962	15.4	0.9	5.844156
5.5 h	61.74	2.5	4.049239	61.84	2.4	3.880983	28.8	1.4	4.861111	17.5	0.8	4.571429	15.78	0.5	3.168568	61.74	0.5	0.809848	15.78	1.1	6.970849
6 h	61.54	2.8	4.549886	61.87	2.3	3.717472	30.9	1.3	4.20712	17.5	0.5	2.857143	16.02	0.7	4.369538	61.54	3	4.874878	16.02	1	6.242197
7 h	61.55	1.7	2.761982	61.9	2.4	3.877221	31.9	1.4	4.388715	17.7	0.8	4.519774	16.14	0.6	3.717472	61.55	1.5	2.437043	16.14	1.3	8.054523

Table S6. Detailed statistical data (Mean, SD, and RSD %) of colorimetric response (Delta E) over time for MRSA and MSSA strains in 6-well microfluidic chips.

**Delta E**	1 µg/mL			2 µg/mL			4 µg/mL			8 µg/mL			0 µg/mL (PC)			32 µg/mL (NC)		
**MRSA**	Mean	SD	RSD (%)	Mean	SD	RSD (%)	Mean	SD	RSD (%)	Mean	SD	RSD (%)	Mean	SD	RSD (%)	Mean	SD	RSD (%)
1 h	5.6	0.5	8.928571	5.5	0.5	9.090909	5.7	0.5	8.77193	5.02	0.2	3.984064	5.4	0.4	7.407407	5.89	0.4	6.791171
2 h	12.04	0.5	4.152824	12.92	0.8	6.19195	12.54	1	7.974482	12.7	0.6	4.724409	12.04	0.5	4.152824	13.01	0.4	3.074558
3 h	13.15	0.7	5.323194	13.2	0.5	3.787879	13.25	1.2	9.056604	13.14	1	7.61035	13.15	0.5	3.802281	13.21	0.8	6.056018
3.5 h	36.4	1.5	4.120879	35.9	1.4	3.899721	36.2	1.3	3.59116	23.2	1.6	6.896552	36.4	1.2	3.296703	15	1.02	6.8
4 h	49.6	2	4.032258	48.9	1.6	3.271984	49.2	2.1	4.268293	35.09	1.5	4.274722	49.6	1.5	3.024194	17.1	1	5.847953
4.5 h	49.5	2	4.040404	48.8	1.8	3.688525	49.02	2.6	5.303958	47.1	2.3	4.883227	49.5	2.1	4.242424	19.2	1.1	5.729167
5 h	61.98	1.8	2.904163	62.01	2.52	4.063861	62.16	2.6	4.182754	55.3	3.7	6.690778	61.98	2.1	3.38819	19.8	0.8	4.040404
5.5 h	62.14	2.8	4.505954	62.18	2.7	4.342232	62.24	1.9	3.052699	56.03	2	3.569516	62.14	1.5	2.413904	19.8	0.9	4.545455
6 h	65.9	3.01	4.567527	66.5	3.4	5.112782	66.46	2.4	3.611195	62.1	2.3	3.703704	65.9	2.4	3.641882	20.1	1.1	5.472637
7 h	68.8	3.05	4.43314	68.4	3.6	5.263158	67.9	3.4	5.007364	64.8	4.6	7.098765	68.8	2.5	3.633721	20.1	0.5	2.487562

**Delta E**	1 µg/mL			2 µg/mL			4 µg/mL			8 µg/mL			0 µg/mL (PC)			32 µg/mL (NC)		
**MSSA**	Mean	SD	RSD (%)	Mean	SD	RSD (%)	Mean	SD	RSD (%)	Mean	SD	RSD (%)	Mean	SD	RSD (%)	Mean	SD	RSD (%)
1 h	5.4	0.5	9.259259	5.52	0.5	9.057971	5.6	0.4	7.142857	5.1	0.4	7.843137	5.4	0.5	9.259259	5.02	0.2	3.984064
2 h	15.04	0.6	3.989362	12.89	0.9	6.982157	8.54	0.7	8.196721	10.7	0.8	7.476636	15.04	0.7	4.654255	9.7	0.4	4.123711
3 h	15.15	0.5	3.30033	13.18	1	7.587253	9.25	0.9	9.72973	11.14	0.8	7.181329	15.15	0.8	5.280528	10.14	0.7	6.903353
3.5 h	39.6	1.2	3.030303	30.4	1.4	4.605263	15.36	1.2	7.8125	15.5	1.1	7.096774	39.6	1.4	3.535354	14.5	1	6.896552
4 h	51.7	2.2	4.255319	42.1	1.9	4.513064	22.3	1.7	7.623318	15.8	1.4	8.860759	51.7	1.5	2.901354	15.1	0.8	5.298013
4.5 h	57.8	2.1	3.633218	43.2	1.3	3.009259	25.5	2	7.843137	16.2	1.1	6.790123	57.8	2.4	4.152249	15.5	1.3	8.387097
5 h	61.79	2.52	4.07833	48.8	2.2	4.508197	28.5	2.52	8.842105	16.2	1.6	9.876543	61.79	1.7	2.751254	16.5	1.3	7.878788
5.5 h	61.74	2.8	4.535147	56.84	3.1	5.453906	29.5	2.83	9.59322	17.9	1.4	7.821229	61.74	2.5	4.049239	16.6	0.9	5.421687
6 h	61.54	3.1	5.037374	58.87	3.7	6.285035	30.9	2.3	7.443366	17.9	1.3	7.26257	61.54	2	3.249919	17.5	0.9	5.142857
7 h	61.55	3.7	6.011373	59.9	3.7	6.176962	32.2	2.9	9.006211	18.1	1.6	8.839779	61.55	2.5	4.061738	17.5	1.1	6.285714

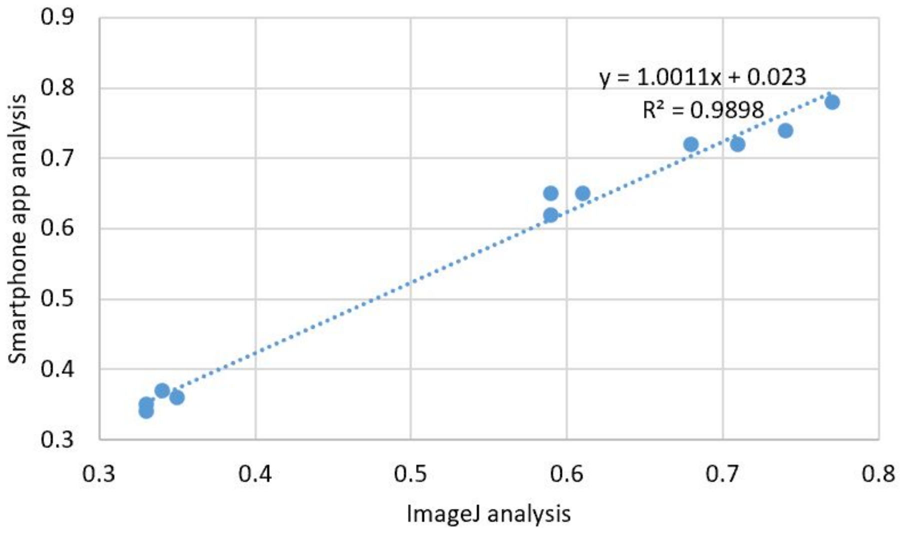

Figure S3. Validation of the smartphone-based image analysis algorithm.
Correlation analysis comparing the R/B intensity ratios of bacterial samples measured by the developed Android application (y-axis) versus standard ImageJ software (x-axis). The linear regression analysis demonstrates a near-perfect agreement (y = 1.0011x + 0.023) with a high correlation coefficient (R^2 = 0.9898), confirming that the smartphone app provides accurate quantitative analysis comparable to benchtop benchmarks.

Table S7. Operational comparison between the proposed microfluidic chip and our prior manual assays.

**Parameter**	**Our Prior Manual Assays (Refs 22, 24, 25)**	**This Work (Microfluidic Chip)**
User Workflow	Serial dilution & multiple pipetting steps	Single-step sample loading
Human Error Risk	High (due to manual volume adjustments)	Negligible (Auto-distributed by fluidics)
Bio-safety	Open-tube handling (Aerosol risk)	Closed-chip system (Safe handling)
Reagent Status	Fresh preparation required	Ready-to-use
Equipment	Test tubes, rack, multiple pipette tips	One chip, one pipette tip
Throughput	Labor-intensive for multi-dose testing	High-throughput potential

Table S8. Comparison of the operational characteristics of the developed smartphone-based microfluidic chip with other recently reported antimicrobial susceptible testing (AST) methods from the literature (2023–2025).

**Detection Principle**	**Platform / Substrate**	**Target Bacteria**	**Time to Result**	**Readout / Equipment**	**Ref.**
Turbidimetry (Standard)	96-well Plate	*S. aureus*	18–24 h	Plate Reader / Spectrophotometer	[4]
CRISPR/Cas9 + SERS	Lateral Flow Strip	*S. aureus*	~20 min	Raman Spectrometer (High Cost)	[5]
Electrical Impedance	Microfluidic Chip	*E. coli / S. aureus*	< 1 h	Impedance Analyzer (Complex)	[6]
Isothermal Amp. (RPA)	Paper-based Device	*S. aureus* (MRSA)	~1 h	Fluorescence Reader	[7]
Machine Learning	Droplet Microfluidics	Multi-species	~3 h	Microscope + PC (GPU)	[8]
Aptamer-Colorimetric	Paper-based Device	*Ampicillin Res.*	~45 min	Smartphone (Reagent heavy)	[9]
Fluorescence + AI	Adaptive Microfluidic Chip	*S. aureus / E. coli*	~1 h	Fluorescence Microscope + PC	[10]
Colorimetric	PMMA Microfluidic Chip	*S. aureus* (MRSA)	4 h	Smartphone (Simple/Low-Cost)	This Work

The comparison reveals that while CRISPR-based [5] and Aptamer-based [9] methods offer the shortest detection times (~20–45 min), they are limited by high equipment costs (e.g., Raman spectrometers for [5]) or complex reagent handling (for [9]). Similarly, other rapid microfluidic approaches [6, 10] necessitate bulky external microscopes or analyzers. The present work distinguishes itself by uniquely combining a generic smartphone readout with simple, stable chemical reagents, offering a balanced solution for resource-limited settings. Abbreviations: SERS, Surface-enhanced Raman scattering; RPA, Recombinase Polymerase Amplification; AI, Artificial Intelligence; *E. coli*, *Escherichia coli*.

References

[1] J. He, M. M. Giusti, Anthocyanins: Natural colorants with health-promoting properties, Annu. Rev. Food Sci. Technol. 1 (2010) 163-187. https://doi.org/10.1146/annurev.food.080708.100754

[2] G. C. Trilini, M. P. Bernardo, D. S. Dias, et al. Intelligent Packaging Systems with Anthocyanin: Influence of Different Polymers and Storage Conditions, Polymers 16(2024), 2886. https://doi.org/10.3390/polym16202886

[3] The European Committee on Antimicrobial Susceptibility Testing, Breakpoint tables for interpretation of MICs and zone diameters Version 16.0. https://www.eucast.org/bacteria/clinical-breakpoints-and-interpretation/clinical-breakpoint-tables/, 2026 (accessed 25 January 2026).

[4] Clinical and Laboratory Standards Institute (CLSI), Methods for Dilution Antimicrobial Susceptibility Tests for Bacteria That Grow Aerobically; Approved Standard—12th Edition. CLSI Document M07-Ed12. Clinical and Laboratory Standards Institute: Wayne, PA. 2024.

[5] J. Wang, H. Jiang, Y. Chen, et al. CRISPR/Cas9-mediated SERS/colorimetric dual-mode lateral flow platform combined with smartphone for rapid and sensitive detection of Staphylococcus aureus, Biosens. Bioelectron. 249 (2024) 116046. https://doi.org/10.1016/j.bios.2024.116046
[6] J. Chen, J. Zhong, Y. Chang, et al. Rapid and Accurate Antimicrobial Susceptibility Testing Using Label-Free Electrical Impedance-Based Microfluidic Platform. Small 20 (2024), 2303352. https://doi.org/10.1002/smll.202303352
[7] W. Chen, J. Zhang, H. Wei, et al. Rapid and sensitive detection of methicillin-resistant Staphylococcus aureus through the RPA-PfAgo system. Front.Microbiol.15 (2024) 1422574. https://doi.org/10.3389/fmicb.2024.1422574
[8] T. Vanhoucke, A. Perima, L. Zolfanelli, et al. Deep learning enabled label-free microfluidic droplet classification for single cell functional assays. Front. Bioeng. Biotechnol. 12 (2024) 1468738. https://doi.org/10.3389/fbioe.2024.1468738
[9] D. Soulis, A. Economou, C. Kokkinos, Smartphone-Addressable Paper-Based Devices for the Colorimetric Detection of Ampicillin Based on Salt-Induced Aggregation of Gold Nanoparticles. Sensors 48 (2023), 40. https://doi.org/10.3390/CSAC2023-14875
[10] Chatzimichail, S.; Turner, P.; Feehily, C.; Farrar, A.; Crook, D.; Andersson, M.; Kapanidis, A. N. Rapid identification of bacterial isolates using microfluidic adaptive channels and multiplexed fluorescence microscopy. Lab on a Chip 24 (2024) 4843–4858. https://doi.org/10.1039/D4LC00325J
